# Correlations between smartphone addiction and alexithymia, attachment style, and subjective well-being: A meta-analysis

**DOI:** 10.3389/fpsyg.2022.971735

**Published:** 2022-09-02

**Authors:** Yueming Ding, Haitao Huang, Yiming Zhang, Qianwen Peng, Jingfen Yu, Guangli Lu, Huifang Wu, Chaoran Chen

**Affiliations:** ^1^School of Nursing and Health, Institute of Nursing and Health, Henan University, Kaifeng, China; ^2^School of Business, Institute of Business Administration, Henan University, Kaifeng, China

**Keywords:** smartphone addiction, alexithymia, attachment, subjective well-being, meta-analysis

## Abstract

**Background:**

Smartphone addiction (SA) has become a social problem that affects peoples’ quality of life and is frequently reported to be correlated with alexithymia, avoidant or anxious attachment styles, and subjective well-being. This study aimed to investigate the relationship between SA and alexithymia, attachment style, and subjective well-being.

**Methods:**

A meta-analysis was conducted following the Preferred Reporting Items for Systematic Reviews and Meta-Analyses (PRISMA) guidelines. The following electronic databases were searched: PubMed, Web of Science, Embase, PsycINFO, PsycArticles, China National Knowledge Infrastructure (CNKI), WANFANG DATA, and Chongqing VIP Information Co., Ltd. (VIP). Stata 16.0 was used to analyze the overall effect and test the moderating effect.

**Results:**

One hundred and ten studies were included, involving a total of 96,680 participants. SA had a significantly high positive correlation with alexithymia (*r* = 0.40), attachment anxiety (*r* = 0.37), and negative emotions (*r* = 0.31), and a low positive correlation with attachment avoidance (*r* = 0.17). In addition, there was a high negative correlation between SA and subjective well-being (*r* = –0.33) and a low negative correlation between SA, life satisfaction (*r* = –0.17), and positive emotions (*r* = –0.18). A moderation analysis revealed that age significantly moderated the relationship between SA and positive emotions. The tools for measuring SA significantly moderated the relationship between SA, alexithymia, attachment anxiety, and subjective well-being. Meanwhile, subjective well-being measurement tools significantly moderated the relationships between SA, subjective well-being, and negative emotions.

**Conclusion:**

SA was closely related to alexithymia, attachment style, and subjective well-being. In the future, longitudinal research can be conducted to better investigate the dynamic changes in the relationship between them.

**Systematic review registration:**

[www.crd.york.ac.uk/PROSPERO/], identifier [CRD42022334798].

## Introduction

With the progress of science and technology and the advancement of digitalization, the emergence of smartphones has not only advanced the global communication industry but also greatly affected people’s lives and behaviors. In September 2021, the number of global smartphone users had reached 3.9 billion, and it is expected that this number will exceed 4.5 billion by 2024 (Newzoo, 2021). As excellent carriers of mobile internet technology, smartphones have been integrated into the daily lives of a large number of people, who use them for online communication, learning, entertainment, and other activities, regarding it as an indispensable necessity. However, the problem is that an increasing number of people use smartphones excessively, and the tendencies for smartphone addiction (SA) are on the rise (Sapacz et al., 2016). SA (also known as “smartphone dependence,” “smartphone overuse,” or “problematic smartphone use”) is defined as a compulsive state in which an individual’s physiological, psychological, and/or social functions are impaired due to the uncontrolled use of smartphones (Chóliz, 2010). Although SA is not specifically acknowledged in the fifth edition of the Diagnostic and Statistical Manual of Mental Disorders (DMS-5) (American Psychiatric Association, 2013) or the eleventh version of the International Classification of Diseases (ICD-11) (World Health Organization [WHO], 2018), many scholars tend to regard SA as a behavioral addiction (Takao et al., 2009; Yen et al., 2009), which manifests in symptoms including tolerance development and withdrawal, subjective loss of control, and functional impairment (Lee et al., 2014; Lin et al., 2016). An increasing amount of evidence has shown that SA not only causes a series of mental health problems, such as anxiety and depression (Coyne et al., 2020), but also damages the physical health of individuals, resulting in visual fatigue, reduced immunity, and sleep disorders (Liu Q. Q. et al., 2017). Simultaneously, it also causes individual cognitive failure (Hong et al., 2020) and has a negative impact on academic achievement, coping styles, and family and other interpersonal relationships (Clayton et al., 2015; Nayak, 2018; Lu et al., 2021). According to mental health experts, SA will become one of the most important behavioral addictions of the twenty-first century (Chóliz, 2010).

In view of the harmful effects of SA on individual physical and mental health, several scholars have actively explored the influential factors of SA and found that alexithymia, anxious and avoidant attachment styles, and subjective well-being are important factors affecting SA (Remondi et al., 2020; Satici and Deniz, 2020; Bermingham et al., 2021; Xiao et al., 2021). Alexithymia refers to the difficulties that an individual encounters when identifying and describing their own and others’ emotions, and distinguishing between feelings and bodily sensations and the externally oriented cognitive styles (Taylor et al., 1999). Over the past few decades, the role of alexithymia in substance use disorders and behavioral addiction has attracted researchers’ interest, and there is increasing evidence that alexithymia may play an important role in the pathogenesis of addictive disorders. For example, alexithymia is significantly positively correlated with the alcohol addiction severity, gambling disorder, and eating disorder (Stasiewicz et al., 2012; Barth, 2016; Estévez et al., 2021; Velotti et al., 2021). According to cognitive-behavioral theory, due to the lack of cognitive ability and emotional defects, alexithymia individuals usually have difficulties in facing and dealing with stressful conditions, and it is difficult to establish and maintain healthy interpersonal relationships. They may overuse smartphones to meet their social needs (ŞahÝn et al., 2009). In line with this, recent research has suggested that alexithymia bears a significant positive relationship with SA (Remondi et al., 2020; Gündoğmuş et al., 2021; Xiao et al., 2021). However, the correlation coefficients of different research results are quite different. For example, some studies have found a moderate positive correlation between them (Gao T. et al., 2018; Yavuz et al., 2019), while others have found a high positive correlation (Kaya, 2021; Xiao et al., 2021).

Attachment perspective has made an important contribution to the understanding of addictive behavior. The current attachment model can be described from two dimensions: attachment anxiety and attachment avoidance (Brennan et al., 1998; Velotti et al., 2022). Attachment anxiety usually refers to individuals who are afraid of interpersonal rejection and eager to stay close to others. It’s stressful when your partner isn’t around. Attachment avoidance refers to individuals who feel uncomfortable and afraid of getting emotional support from others, and are overly dependent on themselves instead of trusting others. Attachment theory points out (Bowlby, 1982) that feelings of perceiving close others as unreliable and untrustworthy seriously threaten attachment security, triggering maladaptative and compensatory reactions, and aim to restore security through other sources. Smartphones represent a tool for maintaining relationships and the storage of social relationships and memories, which makes it an easier target for compensatory attachment than other objects (Konok et al., 2016). Most studies support this view, namely, that attachment styles is significantly associated with SA (Park et al., 2020; Remondi et al., 2020; Parent et al., 2021). However, previous findings regarding the magnitudes and directions of the association between attachment styles and SA are quite mixed. For instance, some studies have found a low positive correlation between SA and attachment anxiety (Remondi et al., 2020), some have found a moderate positive correlation (Liu et al., 2019; Parent et al., 2021), and some studies have found a high positive correlation between them (Han et al., 2017; Park et al., 2020). However, studies on the relationship between SA addiction and attachment avoidance have shown that the correlation properties and coefficients of the two are significantly different. Overall, a few studies have found not only a high (Gui et al., 2021), moderate (Kim and Koh, 2018; Remondi et al., 2020), and low positive correlation (Du et al., 2016; Wang, 2018), but also an insignificant relationship (Park et al., 2020; Parent et al., 2021).

Subjective well-being refers to life satisfaction and positive and negative emotions generated by an individual’s overall evaluation of his life quality based on his own standards (Diener, 2009). According to use-satisfaction theory (Parker and Plank, 2000), individuals with low subjective well-being can temporarily escape from troubles through smartphones, and experience pleasure and relaxation in the process of playing online games, online social networking, etc., which may make them use smartphones more frequently. Additionally, the compensation internet use theory points out that (Kardefelt-Winther, 2014a) individuals with low subjective well-being also have a more negative perception of their relationship with others. They tend to believe that others cannot understand themselves, and use smartphones more to obtain social support, so they are more dependent on smartphones (Ozdemir et al., 2018; Volkmer and Lermer, 2019; Ding et al., 2021). Many studies have revealed that subjective well-being can negatively predict SA. However, empirical findings on the strength of this association are mixed. Specifically, regarding the relationship between SA and subjective well-being, some studies have found a low negative correlation between them (Li et al., 2017; Zhang F. et al., 2020), some have found a moderate negative correlation (Chen et al., 2019; Satici and Deniz, 2020), and a few others have found a high negative correlation between them (Gao et al., 2020; Wang C. et al., 2021). As for the relationship between SA, life satisfaction, and positive emotions, there was a significant difference in the correlation properties and correlation coefficients between them. Overall, not only a high (An et al., 2019; Li, 2019), moderate (Yang L., 2019; Hou et al., 2021), and low negative correlation (Horwood and Anglim, 2019; Yang et al., 2019) was found but also an insignificant relationship was found by a few studies (Md Nordin, 2019; Zhang, 2019). As for the relationship between SA and negative emotions, most studies showed a low to high positive correlation (Gao Y. et al., 2018; Tong et al., 2019; Zhang, 2019), but some studies showed that there was a significant negative correlation between them (Huang, 2021).

To date, there is little consensus on the extent to which alexithymia, attachment styles, and subjective well-being is correlated with SA. Therefore, the first purpose of this study was to explore the relationship between SA and alexithymia, attachment styles, and subjective well-being.

As a secondary goal, we explored the potential moderators of the effect sizes. Age, gender, and measurement tools were considered, as potential moderators. First, individuals of different ages have different psychological needs, social relationships, growth tasks, and social environments. From the perspective of development, alexithymia is a cumulative process that begins in childhood and develops and strengthens as we grow older (Kauhanen et al., 1993). Similarly, during the transition from high school to college, many college students experience a decrease in their subjective well-being owing to changes in their environment (Oswald and Clark, 2003). In addition, a meta-analysis also confirmed the age-specific distinctions in SA (Ran et al., 2022). Specifically, the association between social anxiety and SA was stronger in younger individuals than in older persons. Therefore, the developmental level of alexithymia and subjective well-being at different ages may affect the level of SA.

Second, the study found that SA has a greater inducing effect on alexithymia in women than in men (Zhang et al., 2018b). Men and women may differ in the regulation of the relationship between insecure attachment dimensions and SA (Remondi et al., 2020). In addition, women are found to attach greater importance to social relationships than men (Ying and Dai, 2008) and more often, use smartphones to establish and maintain social relationships (Beranuy et al., 2009). The quality of social relationships has an important impact on the experience of subjective well-being (Tomé et al., 2014). In addition, previous studies have revealed gender differences in the pattern of smartphone use (Volkmer and Lermer, 2019; Su et al., 2020). Therefore, it is necessary to examine the moderating effect of gender.

Finally, the focus of the various measurement tools is different. In terms of the measurement tools of SA, the Mobile Phone Addiction Index (MPAI) (Leung, 2008), the Mobile Phone Addiction Tendency Scale for College Students (MPATS) (Xiong et al., 2012) and the Smartphone Addiction Scale (SAS) (Kwon et al., 2013) are widely used at present. These three measurement tools cover different contents, and the core components of each are also different. Similarly, in terms of measuring attachment, two of the most widely used tools are the Experiences in Close Relationships Inventory (ECR) (Sibley et al., 2005) and the Adult Attachment Scale (AAS) (Collins and Read, 1990). The former divides attachment into two dimensions, attachment anxiety and attachment avoidance, while the latter divides attachment into three dimensions: closeness, dependence, and anxiety. The division into different dimensions may have an impact on the final results. In addition, in terms of the measuring tool of subjective well-being, at present, the scale for this feature discusses overall well-being, life satisfaction (cognitive component of subjective well-being), positive-negative affect, and emotional balance (the emotional component of subjective well-being) from the dimensions of wholeness, cognition, and emotion, respectively. Different research perspectives may lead to different levels of well-being. Although the results measured using the emotional balance method were partially similar to those measured using the life satisfaction scale, they were not the same (Pavot, 2008).

As a whole, although the relationship between alexithymia, attachment style, subjective well-being and SA has attracted increasing attention. However, there has been no consensus on the extent to which these factors are related to SA. In addition, whether these relationships are disturbed by studies characteristics has also become a question that needs further discussion. Therefore, the aims of this meta-analysis were to (1) determine the overall effect size for the relationship between SA and alexithymia, attachment style, and subjective well-being, and (2) examine whether age, gender and measurement tools moderate this relationship.

## Methods

This meta-analysis followed the guidelines of Preferred Reporting Items for Systematic Reviews and Meta-Analyses (PRISMA) (Moher et al., 2009) (see the checklist in Supplementary Material 1) and was registered at PROSPERO (registration number CRD 42022334798).

### Literature search

The PubMed, Web of Science, Embase, PsycINFO, PsycArticles, China National Knowledge Infrastructure (CNKI), WANFANG DATA, and Chongqing VIP Information Co., Ltd. (VIP) databases were searched for eligible studies published up to December 19, 2021. Search terms used for smartphones included “cell phone,” “mobile phone,” “smart phone,” “smartphone,” “cellular phone,” “transportable Cellular Phones,” “portable Cellular Phone,” “Cellular Telephone,” “Mobile Telephone,” and “Car Phone”. Search terms used for addiction included “addiction,” “dependence,” “abuse,” “dependency,” “addicted to,” “overuse,” “problem use,” and “compensatory use”. Search terms used for alexithymia included “Affective Symptom*,” “Symptom*, Affective,” “Alexithymia*,” “Emotional Disturbance*,” and “Disturbance*, Emotional”. Search terms used for attachment included “Attachment,” “Attachment Disorder*, Reactive,” “Disorder*, Reactive Attachment,” and “Reactive Attachment Disorder*”. Search terms used for subjective well-being included “happiness,” “well-being,” “subjective well-being,” “life satisfaction,” “positive emotion,” and “negative emotion”. A detailed search strategy is available in Supplementary Material 2. We also conducted a search of gray literature in Google Scholar. Furthermore, reference lists of retrieved studies were manually reviewed to identify further potentially eligible studies.

### Inclusion and exclusion criteria

The inclusion criteria for the studies were as follows: (a) they were cross-sectional studies; (b) they used a validated scale to assess SA, alexithymia, attachment styles, and subjective well-being; (c) alexithymia measurement instruments were limited to the TAS-20; (d) attachment measurement instruments were limited to the ECR or AAS; (e) the correlation coefficient between SA and alexithymia or attachment styles or subjective well-being was reported, and if the correlation coefficient of the total score was not reported, the full factor correlation coefficient was reported; (f) written in English or Chinese; and (g) both published articles and dissertations were included. The exclusion criteria were as follows: (a) conference abstracts and review articles; (b) studies with the same data published repeatedly; (c) literature was of poor quality; and (d) studies with samples containing individuals with physical diseases or mental disorders.

### Data extraction

All studies were coded independently by two reviewers (YMD and HTH), recording first author and year of publication, country, sample size, proportion of females, age, correlation coefficient, SA scale, alexithymia scale, attachment scale, and subjective well-being scale (see Table 1). For the input of correlation coefficient, there are the following coding standards: (a) If the correlation coefficient between SA and alexithymia, attachment style, or subjective well-being scale is not reported, but the values of *F*, *T*, andχ^2^ are reported, they are transformed into the *r*-value by the corresponding formula (*r* = $\sqrt{\frac{t^{2}}{t^{2}+d\,f}},$*df* = n_1_+n_2_-2; *r* = $\sqrt{\frac{F}{F+d\,f_{e}}};$*r* = $\sqrt{\frac{\chi^{2}}{\chi^{2}+N}}$) (Card, 2015). (b) The study effect size was encoded as an effect size according to the independent samples. If a study contained multiple independent samples, the article effect size was coded separately. (c) If only the correlation coefficients of certain dimensions between SA and alexithymia or attachment style or subjective well-being were reported, the average of each dimension was taken before coding.

**TABLE 1:** Characteristics of the 110 studies included in the meta-analysis.

References	Country	*N*	Female%	Age	MPA measure	Outcome measure	Outcome (R)
Ge et al. (2013)	China	877	32.7	1 and 2	MPATS	ECR	Attachment A (0.47) and Attachment B (–0.01)
Wang (2014)	China	751	58.5	2	MPAI	TAS-20	Alexithymia (0.36)
Huang et al. (2014)	China	1,392	42.7	2	MPAI	GWB	SWB (–0.49)
Ji et al. (2014)	China	163	66.3	2	MPATS	OHI	SWB (–0.28)
Yuan (2014)	China	832	55.3	1	MPAI	ASLSS	SWB (–0.41)
Zhang et al. (2015b)	China	4,147	68.9	2	SQAPMPU	TAS-20	Alexithymia (0.37)
Zeng (2015)	China	282	60.3	2	MPATS	ECR	Attachment A (0.44) and Attachment B (0.03)
Zhang et al. (2015a)	China	1,455	50.4	2	MPAI	ECR/GWB	Attachment A (0.32) and Attachment B (0.15)/SWB (–0.31)
Deng et al. (2015)	China	1,477	43.1	2	MPAI	GWB	SWB (–0.49)
Kan (2015)	China	430	86.3	2	MPAI	SWLS	LS (–0.11)
Tang et al. (2015)	China	966	56.8	2	MPATS	GWB	SWB (–0.28)
Wang and Zhang (2015)	China	3,738	65.7	2	MPAI	SWB	SWB (–0.27) and LS (–0.15) and PE (–0.13) and NE (0.28)
Xie (2015)	China	691	62.7	2	SQAPMPU	PANAS	PE (–0.06) and NE (0.44)
Hou et al. (2016)	China	611	36.8	2	MPATS	TAS-20	Alexithymia (0.43)
Zheng (2016)	China	742	42.6	1	MPAI	TAS-20	Alexithymia (0.54)
Li (2016)	China	1,105	52.2	2	MPAI	TAS-20	Alexithymia (0.33)
Xie (2016)	China	409	47.9	2	MPAI	ECR	Attachment A (0.56) and Attachment B (0.28)
Du et al. (2016)	China	1,014	72.1	2	MPAI	AAS	Attachment A (0.37) and Attachment B (0.11)
Ge (2016)	China	995	16.5	1	MPATS	GWB	SWB (–0.31)
Li et al. (2016)	China	1,620	43.2	2	MPAI	GWB	SWB (–0.46)
Samaha and Hawi (2016)	China	249	45.8	2	SAS-SV	SWLS	LS (0.08)
Sun et al. (2017)	China	684	42.7	2	MPAI	TAS-20	Alexithymia (0.26)
Han et al. (2017)	China	543	59.1	2	MPAI	ECR	Attachment A (0.38)
Arpaci et al. (2017)	Turkey	450	70.9	2	NMP-Q	ECR	Attachment A (0.54) and Attachment B (0.27)
Yuchang et al. (2017)	China	297	45.5	2	SAS-SV	AAS	Attachment A (0.17)
Li (2017)	China	1,507	74.5	2	MPATS	SWLS	LS (0.14)
Li et al. (2017)	China	598	44.6	2	WMPDQ	IWB	SWB (–0.16)
Liu Q. et al. (2017)	China	1,258	46.6	1	MPAI	W’s ABS	SWB (–0.32)
Ouyang (2017)	China	2,502	52.6	2	MPATS	ASLSS	LS (–0.15)
Peng (2017)	China	408	27.7	1	WMPDS	GWB	SWB (–0.17)
Wang (2017)	China	937	53.5	2	MPAI	GWB	SWB (–0.37)
Zhang et al. (2017)	China	359	60.2	2	MPATS	PANAS	PE (–0.08) and NE (0.29)
Hao (2018)	China	1,380	43.8	1	MPAI	TAS-20	Alexithymia (0.30)
Zhang et al. (2018b)	China	472	56.4	2	MPATS	TAS-20	Alexithymia (0.40)
Gao T. et al. (2018)	China	1,105	52.2	2	MPAI	TAS-20	Alexithymia (0.23)
Mei et al. (2018)	China	1,034	52.7	2	MPAI	TAS-20	Alexithymia (0.35)
Huang (2018)	China	352	67.1	2	MPAI	ECR	Attachment A (0.25) and Attachment B (0.09)
Wang (2018)	China	346	61.1	2	WMPDQ	ECR	Attachment A (0.43) and Attachment B (0.18)
Kim and Koh (2018)	Korea	313	58.1	2	APS-A	ECR-R	Attachment B (0.24)
Gao Y. et al. (2018)	China	360	53.9	2	MPAI	PANAS	PE (–0.12) and NE (0.25)
Niu et al. (2018)	China	2,394	43.9	2	MPAI	GWB	SWB (–0.48)
Ren (2018)	China	628	73.9	2	MPAI	PANAS	PE (–0.19) and NE (0.41)
Xiong et al. (2018)	China	359	60.2	2	MPATS	NSA	NE (0.29)
Yang (2018)	China	1,040	58.9	2	MPAI	PANAS	NE (0.30)
Zhang et al. (2018a)	China	732	59.6	2	MPAI	PANAS	NE (0.35)
Zufeiya and Li (2018)	China	1,764	48.3	2	MPAI	GWB	SWB (–0.46)
Ozdemir et al. (2018)	Pakistanand Turkey	729	70.6	2	NMP-Q	A’ SHS	SWB (–0.57)
Aruna (2019)	China	519	33.5	2	MPAI	TAS-20	Alexithymia (0.27)
Chen and Shao (2019)	China	547	69.7	2	MPATS	TAS-20	Alexithymia (0.39)
Huang et al. (2019)	China	479	64.9	2	MPATS	TAS-20	Alexithymia (0.48)
Lin (2019)	China	453	46.6	1	MPAI	TAS-20	Alexithymia (0.56)
Li and Hao (2019)	China	693	46.5	1	MPAI	TAS-20	Alexithymia (0.38)
Hao et al. (2019)	China	847	48.8	2	MPAI	TAS-20	Alexithymia (0.34)
Yavuz et al. (2019)	Turkey	1,807	54.0	1	NMP-Q	TAS-20	Alexithymia (0.23)
Xu and Zhou (2019)	China	418	62.7	2	MPATS	ECR	Attachment A (0.45) and Attachment B (0.11)
Yan (2019)	China	426	60.6	2	MPAI	AAS	Attachment A (0.24)
Zhu et al. (2019)	China	755	60.5	2	MPAI	ECR	Attachment A (0.37) and Attachment B (0.18)
Liu et al. (2019)	China	908	52.2	2	MPAI	ECR	Attachment A (0.28)
An et al. (2019)	China	332	60.5	2	MPATS	SWLS/PANAS	LS (–0.15) and PE (–0.39) and NE (0.36)
Chen et al. (2019)	China	1,912	63.2	2	MPAI	SWLS/PANAS	SWB (–0.23)
Li (2019)	China	380	54.5	2	MPATS	SWB	SWB (–0.44) and LS (–0.61) and PE (–0.24) and NE (0.22)
Tong et al. (2019)	China	1,162	54.6	2	MPAI	PANAS	NE (0.35)
Yang L. (2019)	China	615	63.3	2	SAS-C	PANAS	PE (–0.20) and NE (0.27)
Yang Z. (2019)	China	730	49.0	1	MPPUS-10	ASLSS	LS (–0.34)
Zhang (2019)	China	328	52.1	1	WMPDS	ASLSS	SWB (–0.12) and LS (–0.15) and PE (0.09) and NE (0.17)
Zhao (2019)	China	651	74.4	2	SAS-C	CSSWBS	SWB (–0.23)
Horwood and Anglim (2019)	Australia	539	79.0	2	MPPUS	SWLS/PANAS	LS (–0.06) and PE (–0.19) and NE (0.31)
Md Nordin (2019)	Malaysia	303	60.4	2	SAS	SWLS	LS (–0.08)
Song et al. (2019)	Korea	328	100.0	3	SAS	SWLS	LS (–0.11)
Volkmer and Lermer (2019)	NR	461	71.4	2 and 3	TMDbrief	WHO-5/SWLS	SWB (–0.23)/LS (–0.12)
Yang et al. (2019)	China	475	44.0	2	SAS-SV	SWLS	LS (–0.16)
Eksi et al. (2020)	Turkey	337	49.0		SABAS	EPOCH	SWB (–0.15)
Huang and Zhao (2020)	China	1,224	44.3	2	MPATS	TAS-20	Alexithymia (0.55)
Yu et al. (2020)	China	1,081	69.2	2	MPATS	TAS-20	Alexithymia (0.57)
Yu and Yu (2020)	China	918	68.6	2	MPATS	TAS-20	Alexithymia (0.55)
Yuan (2020)	China	870	77.0	2	TMD-C	TAS-20	Alexithymia (0.35)
Elkholy et al. (2020)	China	200	57.5	2	SAS-SV	TAS-20	Alexithymia (0.38)
Hao and Jin (2020)	China	901	47.5	2	MPAI	TAS-20	Alexithymia (0.34)
Hao et al. (2020)	China	674	49.0	2	MPAI	TAS-20	Alexithymia (0.26)
Remondi et al. (2020)	Italy	539	70.1	1 and 2	SAS-SV	TAS-20/ECR-12	Alexithymia (0.44)/Attachment A (0.16) and Attachment B (0.24)
Park et al. (2020)	Korea	235	68.1	2	SAPS	ECR-K	Attachment A (0.46) and Attachment B (0.09)
Li et al. (2020)	China	345	62.9	2	MPAI	AAS	Attachment A (0.41) and Attachment B (0.20)
Chen and Xiao (2020)	China	512	51.8	2	MPATS	PANAS	NE (0.61)
Hu et al. (2020)	China	504	56.7	2	MPATS	GWB	SWB (–0.33)
Liang et al. (2020)	China	712	77.0	2	MPAI	SWLS	LS (–0.19)
Liu (2020)	China	525	71.4	2	SAS-CA	IWB	SWB (–0.17)
Xiao (2020)	China	452	57.5	1	MPATS	NAS	NE (0.37)
Zhang F. et al. (2020)	China	910	46.5	2	SAS-C	MHQ	SWB (–0.11)
Zhang Y. et al. (2020)	China	1,953	42.4	2	MPAI	GWB	SWB (–0.46)
Jeong et al. (2020)	Korea	768	42.3	1	K-SAS	SWLS	LS (–0.28)
Peng et al. (2020)	China	1,912	63.2	1	MPAI	SWLS	LS (–0.11)
Gao et al. (2020)	China	1,767	46.9	1	MPAI	ISLQ	SWB (–0.39)
Kaya et al. (2020)	Turkey	690	66.7	2	SPAS-SF	OHI	SWB (–0.10)
Satici and Deniz (2020)	Turkey	320	52.2	2	SAS-SV	SHS	SWB (–0.28)
Hou et al. (2021)	China	1,028	70.1	2	MPATS	TAS-20/ASLSS	Alexithymia (0.55)/LS (–0.28)
Sun (2021)	China	1,014	46.6	1 and 2	MPAI	TAS-20/AAS	Alexithymia (0.47)/ Attachment A (0.36)
Zhang (2021)	China	3,090	61.2	2	MPATS	TAS-20	Alexithymia (0.36)
Gündoğmuş et al. (2021)	Turkish	935	54.4	2	SAS-SV	TAS-20	Alexithymia (0.40)
Kaya (2021)	Istanbul	460	54.6	1	SAS-SV	TAS-20	Alexithymia (0.40)
Xiao et al. (2021)	China	1,267	59.2	2	MPAI	TAS-20	Alexithymia (0.40)
Zhang et al. (2021)	China	1,062	60.3	2	MPATS	TAS-20	Alexithymia (0.40)
Gui et al. (2021)	China	784	69.0	2	MPATS	ECR	Attachment A (0.51) and Attachment B (0.43)
Yao and Zhao (2021)	China	439	51.9	2	MPAI	AAS	Attachment A (0.34)
Bermingham et al. (2021)	US	181	80.1	2	MPPUS	ECR-SF	Attachment A (0.30)
Parent et al. (2021)	Canada	375	76.6	2	PMPUS	ECR-R	Attachment A (0.28) and Attachment B (–0.00)
Huang (2021)	China	1,200	53.6	2	MPATS	D’SWB	SWB (–0.39) and LS (–0.30) and PE (–0.37) and NE (–0.09)
Ding et al. (2021)	China	1,725	57.1	2	MPATS	IWBS-cr	SWB (–0.28)
Li et al. (2021)	China	941	50.6	1	SAI	GWB	SWB (–0.39)
Wang C. et al. (2021)	China	496	61.5	2	MPAI	SWLS	SWB (–0.43)
Wang (2014)	China	769	81.0	2	MPAI	PANAS	NE (0.31)

1, Adolescent; 2, Undergraduate; 3, Non-student group (age over 24 years old); NR, Not Reported; MPATS, Mobile Phone Addiction Tendency Scale for College Students; MPAI, Mobile Phone Addiction Index; SQAPMPU, Self-rating Questionnaire for Adolescent Problematic Mobile Phone Use; SAS-SV, Smartphone Addiction Scale-Short Version; NMP-Q, The Nomophobia Questionnaire; WMPDQ, Wang’s Mobile Phone Dependence Questionnaire for College Students; WMPDS, Wang’s Mobile Phone Dependency Scale for Middle School Students; APS-A, the Smartphone Addiction Proneness Scale for Adult; SAS-C, Smartphone Addiction Scale for College Students; MPPUS-10, a short version of the Mobile Phone Problem Use Scale; MPPUS, the Mobile Phone Problem Use Scale; SAS, Smartphone Addiction Scale; TMD brief, the brief version of the Test of Mobile Phone Dependence; SABAS, the Smartphone Application-Based Addiction Scale; TMD-C, The Test of Mobile Phone Dependence for Chinese adolescents; SAPS, Smartphone Addiction Proneness Scale; SAS-CA, the Smartphone Addiction Scale for Chinese Adults; K-SAS, the Korean Smartphone Addiction Proneness Scale for Youth and Adults; SPAS-SF, the Smart Phone Addiction Scale Short Form; PMPUS, the Problematic Mobile Phone Use Scale; SAI, the Smartphone Addiction Index; TAS-20, the twenty-item Toronto alexithymia scale; ECR, the Experience in Close Relationships Scale; GWB, General Well-Being; OHI, Oxford Happiness Inventory; ASLSS, Adolescent student life satisfaction scale; SWLS, Satisfaction With Life Scale; SWB, Subject Well-Being Scale; PANAS, the Positive and Negative Affect Scale; AAS, the Adult Attachment Scale; IWB, Index of Well-Being; W’s ABS, Wang’s Affect Balance Scale; ECR-R, the Experiences in Close Relationships-Revised-Korean; A’ SHS, Akin’s Self-Happiness Scale; CSSWBS, College Student Subjective Well-Being Scale; MHQ, Multiple Happiness Questionnaire; ISLQ, Inventory of Subjective Life Quality; SHS, The Subjective Happiness scale; ECR-SF, Experience of Close Relationships Scale-Short Form; ECR-R, the revised version of the Experience in Close Relationships Scale; D’SWB, Diener’s Subject Well-Being Scale; IWBS-cr, the Index of Well-Being Scale-China Revised; WHO-5, the WHO-Five well-being index; EPOCH, the EPOCH Measure of Adolescent Well-Being; Attachment A, Attachment anxiety; Attachment B, Attachment avoidance; PE, Positive emotion; NE, Negative emotion.

### Quality assessment

The quality of the studies was assessed independently by two reviewers (YMD and HTH). Any doubts or disagreements were resolved by consulting a third researcher (CRC). The methodological quality of the included studies was assessed by using the nine-item Joanna Briggs Institution Critical Appraisal Checklist for Studies Reporting Prevalence Data (Munn et al., 2015). The score for each item is zero (“no,” “unclear” or “not applicable”) or one (“Yes”), and the highest score is nine. Higher scores reflected better methodological quality.

### Statistical analysis

Stata 16.0 was used for meta-analysis, and effect sizes were calculated as correlations (*r*) in this study. Specifically, the correlations (*r*) were first converted to the corresponding Fisher’s *Z*-value by using the Fisher transform, weighted based on the sample size with 95% confidence intervals: Z = 0.5*ln[(1+r)/(1-r)], where the variance of Z is V*_*Z*_* = 1/n-3 and the standard deviation of Z is SE*_*Z*_* = square root of (1/n-3). The degree of association was interpreted through Gignac and Szodorai’s criteria with effects of 0.10 deemed small, 0.20 deemed moderate, and equal to and larger than 0.30 interpreted as high (Gignac and Szodorai, 2016). Publication bias was analyzed by funnel plots and Egger’s linear regression test, and the Cochran’s Q and *I*^2^ statistics were used to assess heterogeneity. When the *Q*-value was significant (*p* < 0.05) and *I*^2^≥ 75%, this indicated a high degree of heterogeneity in the study, and thus, the random effects model was used; otherwise, the fixed effects model was chosen (Huedo-Medina et al., 2006). In addition, subgroup analysis and sensitivity analysis were conducted to investigate the sources of heterogeneity.

## Results

### Characteristics of the included studies and quality assessment

**FIGURE 1:**
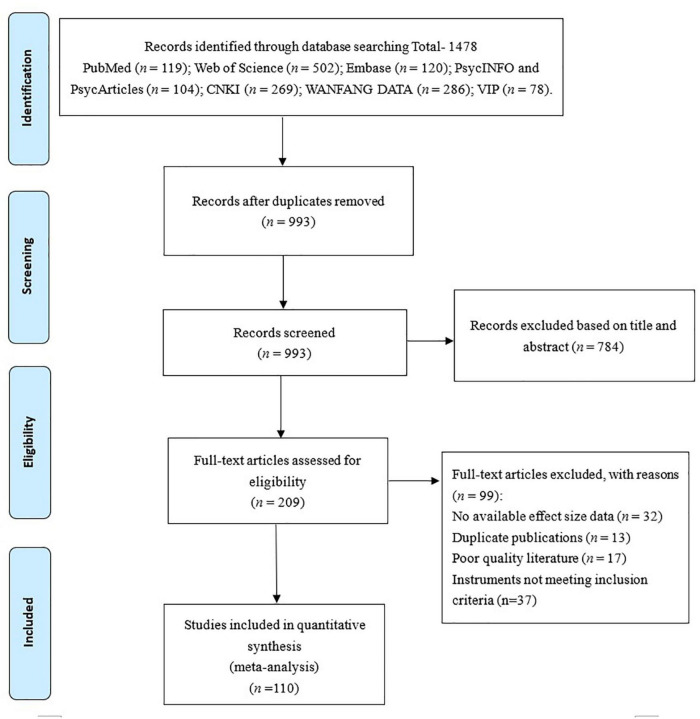
The flow chart of the study selection process.

The initial search yielded 1,478 studies. Duplicate records (*n* = 485) were removed, and 784 studies were excluded based on their titles and abstracts. The full texts of the 209 remaining papers were reviewed, and 110 studies were finally included (see Figure 1), which were published between 2013 and 2021. Collectively 96,680 participants were enrolled in the included studies, most of whom were recruited from schools, with participant numbers ranging from 163 to 4,147 per study. Of the 93,379 participants whose gender was reported, 55.3% were female. Participants were from several different countries across the world: 91 samples were from China, 8 from Turkey, 4 from Korea, 1 from Malaysia, 1 from Egypt, 1 from Australian, 1 from Italy, 1 from the US, and 1 from Canada (see Table 1). In general, the quality of the included studies was either medium or high. Detailed information regarding the quality assessment of each study can be found in Supplementary Material 3.

### Effect size and heterogeneity test

A heterogeneity test was conducted on the included effect sizes, and the results showed that the *Q*-values of alexithymia, attachment anxiety, attachment avoidance, subjective well-being, life satisfaction, positive emotions, and negative emotions were 430.02 (*p* < 0.001), 167.50 (*p* < 0.001), 136.10 (*p* < 0.001), 627.64 (*p* < 0.001), 363.38 (*p* < 0.001), 120.47 (*p* < 0.001), and 318.57 (*p* < 0.001), respectively, and the *I*^2^*-*values were 92.6, 87.5, 89.0, 94.9, 95.0, 91.7, and 94.7%, respectively, both higher than the 75% rule proposed by Higgins et al. (2003), indicating a high level of heterogeneity among the studies. Therefore, the random effects model was selected for the meta-analysis. The results also suggest that it is necessary to explore the moderating variables that affect the relationship between them.

The random effects model showed a high positive correlation between SA addiction and alexithymia, attachment anxiety, and negative emotions and a low positive correlation between SA and attachment avoidance. In addition, there was a high negative correlation between SA and subjective well-being, and a low negative correlation between SA, life satisfaction, and positive emotions (alexithymia: *r* = 0.40, 95% CI = 0.36 to 0.43, *p* < 0.001; attachment anxiety: *r* = 0.37, 95% CI = 0.33–0.42, *p* < 0.001; attachment avoidance: *r* = 0.17, 95% CI = 0.10–0.23, *p* < 0.001; subjective well-being: *r* = –0.33, 95% CI = –0.37 to –0.29, *p* < 0.001; life satisfaction: *r* = –0.17, 95% CI = –0.24 to –0.10, *p* < 0.001; positive emotions: *r* = –0.18, 95% CI = –0.25 to –0.10, *p* < 0.001; and negative emotions: *r* = 0.31, 95% CI = 0.24–0.38, *p* < 0.001) (Table 2).

**TABLE 2:** Effect size and its heterogeneity test and publication bias test.

Outcome variable	*k*	*N*	*r*	95% CI for r	Heterogeneity test			Publication bias test			
					*Q*	*df*	*I*^2^ (%)	Egger’s intercept	*SE*	95%CI	*P*
Alexithymia	33	33,332	0.40	[0.36, 0.43]	430.02***	32	92.6	2.30	2.14	[–2.07, 6.67]	0.29
Attachment anxiety	22	12,444	0.37	[0.33, 0.42]	167.50***	21	87.5	0.40	2.35	[–4.50, 5.31]	0.87
Attachment avoidance	16	8,949	0.17	[0.10, 0.23]	136.10***	15	89.0	–0.17	2.97	[–6.55, 6.20]	0.96
Subjective well-being	33	35,826	–0.33	[–0.37, –0.29]	627.64***	32	94.9	3.85	2.33	[–0.91, 8.61]	0.11
Life satisfaction	19	17,922	–0.17	[–0.24, –0.10]	363.38***	18	95.0	–1.64	2.65	[–7.23, 3.95]	0.55
Positive emotion	11	9,170	–0.18	[–0.25, –0.10]	120.47***	10	91.7	–0.45	2.64	[–6.43, 5.52]	0.87
Negative emotion	18	14,196	0.31	[0.24, 0.38]	318.57***	17	94.7	2.44	2.90	[–3.71, 8.59]	0.41

***p < 0.001.

### Moderator analysis

The heterogeneity of effects across studies was explored through moderator analysis. Subgroup analysis and meta-regression analysis were used to examine the moderating effects of categorical variables (age, tools for measuring SA, tools for measuring attachment and tools for measuring subjective well-being) and continuous variables (gender), respectively.

As shown in Tables 3, 4, the SA measurement tools significantly moderated the relationship between SA and alexithymia (*p* < 0.05). In the tools for measuring SA, the correlation was largest when SA was measured with MPATS (*r* = 0.51, 95% CI = 0.44–0.59), smaller with SAS (*r* = 0.43, 95% CI = 0.39–0.48) and smallest with MPAI (*r* = 0.38, 95% CI = 0.33–0.43) or other scales (*r* = 0.33, 95% CI = 0.22–0.43). However, the moderating effects of age and gender were not significant (all *p* > 0.05).

**TABLE 3:** Subgroup analyses of the summary correlation between SA and alexithymia.

Moderators	*k*	*N*	*r*	95%CI	Between-group effect (*Q*_*BET*_)	*I*^2^ (%)	*P*
**Age**					0.03		0.868
Middle school student	7	6,228	0.43	[0.31, 0.54]		94.7	
Undergraduate	24	25,551	0.42	[0.37, 0.46]		92.2	
**SA measurement**					11.31*		0.010
MPATS	10	10,512	0.51	[0.44, 0.59]		93.0	
MPAI	16	13,862	0.38	[0.33, 0.43]		89.3	
SAS/SAS-SV	4	2,134	0.43	[0.39, 0.48]		0.0	
Others	3	6,824	0.33	[0.22, 0.43]		93.7	

*p < 0.05.

**TABLE 4:** Univariate regression analysis of continuous variables (random effect model).

Moderators		*k*	*SE*	*t*	95%CI	*P*
Female (%)	Alexithymia	33	0.00	1.43	[–0.00, 0.01]	0.16
	Attachment anxiety	22	0.00	–0.37	[–0.01, 0.00]	0.72
	Attachment avoidance	16	0.00	0.74	[–0.00, 0.01]	0.47
	Subjective well-being	33	0.00	0.81	[–0.00, 0.01]	0.43
	Life satisfaction	19	0.00	1.27	[–0.00, 0.01]	0.22
	Positive emotion	11	0.01	–0.15	[–0.01, 0.01]	0.88
	Negative emotion	18	0.00	0.48	[–0.01, 0.01]	0.64

For the relationship between SA and attachment anxiety, the tools for measuring SA played a significant moderating role (*p* < 0.01; *p* < 0.001, respectively). In terms of the tools for measuring SA, the correlation was largest when SA was measured using MPATS (*r* = 0.52, 95% CI = 0.48–0.56), smaller with other scales (*r* = 0.43, 95% CI = 0.31–0.56), and smallest when using MPAI (*r* = 0.37, 95% CI = 0.32–0.42) or SAS (*r* = 0.17, 95% CI = 0.10–0.23). However, gender, and the tools for measuring attachment did not moderate the relationship between SA and attachment anxiety (all *p* > 0.05) (Tables 4, 5).

**TABLE 5:** Subgroup analyses of the summary correlation between SA and attachment.

Moderators	*k*	*N*	*r*	95%CI	Between-group effect (*Q*_*BET*_)	*I*^2^ (%)	*P*
**Attachment anxiety**							
SA measurement					79.40***		0.000
MPATS	4	2,361	0.52	[0.48, 0.56]		0.0	
MPAI	11	7,660	0.37	[0.32, 0.42]		79.6	
SAS/SAS-SV	2	8,36	0.17	[0.10, 0.23]		0.0	
Others	5	1,587	0.43	[0.31, 0.56]		83.9	
Attachment measurement					2.79		0.095
ECR	16	8,909	0.41	[0.35, 0.48]		89.4	
AAS	6	3,535	0.33	[0.27, 0.40]		73.1	
Attachment avoidance							
SA measurement					2.95		0.400
MPATS	4	2,361	0.15	[–0.09, 0.39]		97.0	
MPAI	6	4,330	0.17	[0.12, 0.21]		58.2	
SAS/SAS-SV	1	539	0.25	[0.16, 0.33]		N/A	
Others	5	1,719	0.16	[0.05, 0.26]		79.1	
Attachment measurement					0.13		0.722
ECR	14	7,590	0.17	[0.09, 0.24]		90.1	
AAS	2	1,359	0.15	[0.05, 0.24]		60.4	

***p < 0.001.

For the relationship between SA and attachment avoidance, the subgroup analyses using gender, tools for measuring SA, and tools for measuring attachment did not differ between subgroups (all *p* > 0.05) (Tables 4, 5).

For the relationship between SA and subjective well-being, the tools for measuring SA and subjective well-being played a significant moderating role (*p* < 0.01, *p* < 0.001, respectively). In terms of the tools for measuring SA, the correlation was largest when SA was measured with MPAI (*r* = –0.42, 95% CI = –0.48 to –0.36), smaller with MPATS (*r* = –0.34, 95% CI = –0.39 to –0.29), and smallest with SAS (*r* = –0.29, 95% CI = –0.40 to –0.18) or other scales (*r* = –0.23, 95% CI = –0.34 to –0.12). In terms of the tools for measuring subjective well-being, the correlation was largest when subjective well-being was measured with GWB (*r* = –0.41, 95% CI = –0.47 to –0.36), smaller with other scales (*r* = –0.32, 95% CI = –0.38 to –0.26) and smallest with OHI (*r* = –0.18, 95% CI = –0.36 to 0.00) or IWB (*r* = –0.17, 95% CI = –0.22 to –0.11). However, age and gender did not moderate the relationship between SA and subjective well-being (both *p* > 0.05) (Tables 4, 6).

**TABLE 6:** Subgroup analyses of the summary correlation between SA and subjective well-being.

Moderators	*k*	*N*	*r*	95%CI	Between-group effect (*Q*_*BET*_)	*I*^2^ (%)	*P*
**Subjective well-being**							
Age					1.34		0.247
Middle school student	8	6,866	–0.30	[–0.38, –0.23]		88.5	
Undergraduate	24	28,499	–0.36	[–0.42, –0.30]		95.8	
SA measurement					11.94**		0.008
MPATS	7	5,933	–0.34	[–0.39, –0.29]		70.4	
MPAI	14	22,995	–0.42	[–0.48, –0.36]		95.1	
SAS/SAS-SV	1	320	–0.29	[–0.40, –0.18]		N/A	
Others	11	6,578	–0.23	[–0.34, –0.12]		95.0	
SWB measurement					38.82***		0.000
GWB	13	16,806	–0.41	[–0.47, –0.36]		92.2	
OHI	2	853	–0.18	[–0.36, 0.00]		77.8	
IWB	2	1,123	–0.17	[–0.22, –0.11]		0.0	
Others	16	17,044	–0.32	[–0.38, –0.26]		93.5	
Life satisfaction							
Age					0.58		0.448
Middle school student	4	3,738	–0.23	[–0.35, –0.10]		92.5	
Undergraduate	13	13,395	–0.17	[–0.26, –0.07]		96.2	
SA measurement					3.84		0.280
MPATS	6	6,949	–0.24	[–0.43, –0.05]		98.3	
MPAI	4	6,792	–0.14	[–0.17, –0.11]		30.7	
SAS/SAS-SV	4	1,355	–0.07	[–0.17, 0.02]		68.2	
Others	5	2,826	–0.20	[–0.31, –0.08]		89.2	
LS measurement					6.61		0.086
SWLS	12	8,016	–0.10	[–0.18, –0.02]		91.6	
ASLSS	3	4,260	–0.26	[–0.39, –0.13]		93.4	
SWB	2	4,118	–0.43	[–0.97, 0.12]		99.0	
Others	2	1,528	–0.24	[–0.40, –0.08]		85.6	
Positive emotion							
Age					18.92***		0.000
Middle school student	1	328	0.09	[–0.02, 0.20]		N/A	
Undergraduate	10	8,842	–0.20	[–0.28, –0.13]		90.7	
SA measurement					4.40		0.111
MPATS	4	2,271	–0.28	[–0.43, –0.14]		90.3	
MPAI	3	4,726	–0.14	[–0.17, –0.11]		5.9	
Others	4	2,173	–0.10	[–0.22, 0.03]		87.6	
PE measurement					0.01		0.993
PANAS	7	3,524	–0.18	[–0.26, –0.10]		81.7	
SWB	2	4,118	–0.18	[–0.28, –0.07]		76.7	
Others	2	1,528	–0.15	[–0.62, 0.32]		98.3	
Negative emotion							
Age					0.13		0.714
Middle school student	2	780	0.28	[0.07, 0.50]		88.8	
Undergraduate	16	13,416	0.33	[0.25, 0.41]		95.2	
SA measurement					0.12		0.942
MPATS	7	3,594	0.32	[0.09, 0.54]		97.7	
MPAI	7	8,429	0.33	[0.29, 0.37]		66.0	
Others	4	2,173	0.31	[0.19, 0.43]		87.5	
NE measurement					10.76**		0.005
PANAS	14	8,550	0.37	[0.32, 0.43]		85.4	
SWB	2	4,118	0.27	[0.21, 0.33]		37.3	
Others	2	1,528	0.04	[–0.21, 0.29]		94.1	

**p < 0.01, ***p < 0.001.

For the relationship between SA and life satisfaction, the subgroup analyses using age, gender, the tools for measuring SA, and the tools for measuring life satisfaction did not differ between subgroups (all *p* > 0.05) (Tables 4, 6).

Age played a significant moderating role in the relationship between SA and positive emotions (*p* < 0.001). The correlation for undergraduates (*r* = –0.20, 95% CI = –0.28 to –0.13) was significantly higher than that for middle school students (*r* = 0.09, 95% CI = –0.02 to 0.20). However, gender, the tools for measuring SA, and the tools for measuring positive emotions did not moderate the relationship between SA and positive emotions (all *p* > 0.05) (Tables 4, 6).

The tools for measuring negative emotions played a significant moderating role in the relationship between SA and negative emotions (*p* < 0.01). The correlation was largest when negative emotions was measured with PANAS (*r* = 0.37, 95% CI = 0.32–0.43), smaller with SWB (*r* = 0.27, 95% CI = 0.21–0.33) and smallest with other scales (*r* = 0.04, 95% CI = –0.21 to 0.29). However, age, gender, and the tools for measuring SA did not moderate the relationship between SA and negative emotions (all *p* > 0.05) (Tables 4, 6).

### Publication bias

**FIGURE 2:**
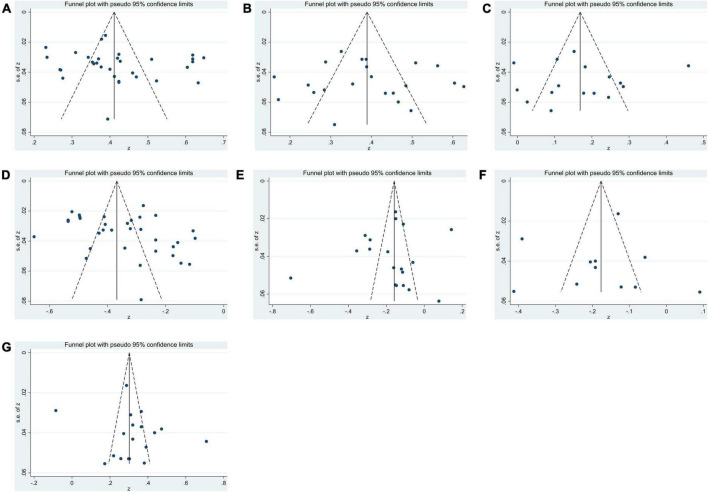
Funnel plots for assessing publication bias within studies related to **(A)** alexithymia, **(B)** attachment anxiety, **(C)** attachment avoidance, **(D)** subjective well-being, **(E)** life satisfaction, **(F)** positive emotion, **(G)** negative emotion.

Publication bias was detected using funnel plots and Egger’s linear regression test. First, Figure 2 shows that the effect sizes of the relationship between SA and alexithymia, attachment anxiety, attachment avoidance, subjective well-being, life satisfaction, positive emotions, and negative emotions were mostly evenly distributed on both sides of the overall effect size, indicating that the risk of publication bias was small in this study. Moreover, Egger’s linear regression tests showed that the *p*-values for alexithymia (*p* = 0.29), attachment anxiety (*p* = 0.87), attachment avoidance (*p* = 0.96), subjective well-being (*p* = 0.20), life satisfaction (*p* = 0.60), positive emotions (*p* = 0.74), and negative emotions (*p* = 0.69) were all greater than 0.05, which further indicated that there was no publication bias in this study, and the estimated results of the meta-analysis were relatively reliable (Table 2).

### Sensitivity analysis

**FIGURE 3:**
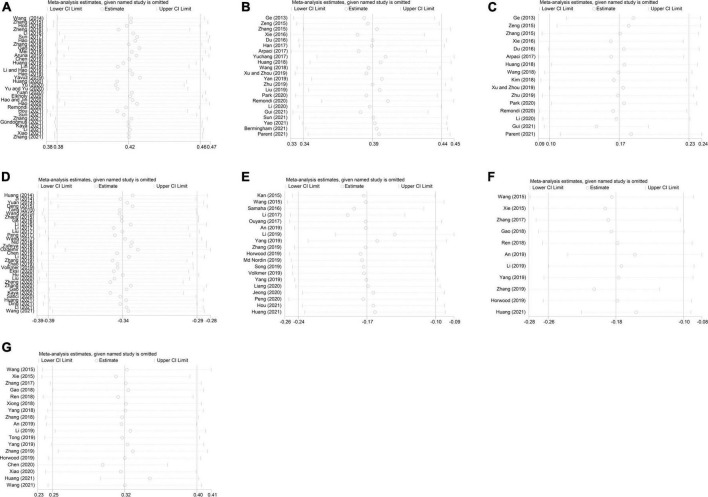
Sensitivity analysis of the correlation between mobile phone addiction and **(A)** alexithymia, **(B)** attachment anxiety, **(C)** attachment avoidance, **(D)** subjective well-being, **(E)** life satisfaction, **(F)** positive emotion, **(G)** negative emotion. Reproduced with permission from Stata 16.0.

To evaluate the robustness of our findings, we used the one-by-one elimination method for sensitivity analysis. Overall, the results were not significantly changed, suggesting that the results of this study were relatively stable (Figure 3).

## Discussion

### Relationship between SA and alexithymia, attachment style, subjective well-being

The results showed high to weak positive correlations between SA and alexithymia, attachment anxiety, negative emotions, and attachment avoidance, with a series of Pearson’s correlation coefficients of 0.40, 0.37, 0.31, and 0.17, respectively. Conversely, there were high to weak negative correlations between SA and subjective well-being, life satisfaction, and positive emotions, with a series of Pearson’s correlation coefficients of –0.33, –0.17, and –0.18, respectively. Importantly, the results from the sensitivity analysis and analyses of publication bias showed that these results were quite robust.

Consistent with previous studies, alexithymia was positively correlated with SA. Individuals with high levels of SA tend to have heavy personal awareness in real life, which makes them indifferent to the expression of emotion in real situations, and they also do not care about feedback and evaluation from the outside world (Zhang et al., 2018b). If the SA trend is not curbed, it may get more difficult for them to communicate realistically with others, and they may find it harder to express emotions properly. Another explanation could be that because individuals with a high degree of alexithymia have a certain cognitive bias in the expression and recognition of emotions (Besharat, 2010), resulting in poor interpersonal adaptability (Hesse and Floyd, 2008). The powerful networks of smartphones provide great opportunities for people to communicate with each other. People tend to establish contact with the outside world through mobile networks and other media, obtain a sense of intimacy, and gradually rely completely on their smartphones to meet all their social needs.

The meta-analysis showed a correlation between SA and insecure attachment styles, which is consistent with previous studies. Specifically, SA is highly positively correlated with attachment anxiety and weakly positively correlated with attachment avoidance. Insecure attachment may lead to difficulty in identifying emotions, poor self-control, and psychological distress (Remondi et al., 2020). According to compensatory Internet use theory, individuals with insecure attachment must find ways (such as surfing the Internet) to release their negative emotions (Kardefelt-Winther, 2014a), which also increases the likelihood of SA due to the convenience of using smartphones to surf the Internet in daily life. It is worth noting that the roles of attachment anxiety and attachment avoidance in predicting individual SA are not equal, and attachment anxiety can better predict SA. This may be related to the over-activation strategy of anxious attachment individuals, that is, they tend to strengthen negative emotional states, exaggerate the threat of the stimulus, and excessively pursue intimate relationships (Ein-Dor et al., 2011). Individuals with attachment anxiety tend to satisfy their needs through virtual worlds constructed using smartphones. Individuals with attachment avoidance interact less in cyberspace because of distrust and neglect by others (Kim and Koh, 2018; Remondi et al., 2020). Therefore, their SA level was lower than that in individuals with attachment anxiety.

The results of the meta-analysis showed that SA was highly negatively correlated with subjective well-being, weakly negatively correlated with life satisfaction and positive emotions, and highly positively correlated with negative emotions. It shows that individuals with SA have lower subjective well-being, life satisfaction, and positive emotions, but higher negative emotions, which is consistent with most previous studies (Wang and Zhang, 2015; Yang et al., 2019; Li et al., 2021). This may be because individuals with low subjective well-being received less social support in real life, while the online social support provided by smartphones can compensate for the lack of social support in real life and help them escape the pain of the real world (Gao et al., 2020). In addition, individuals with low subjective well-being tend to have negative emotional experiences such as anxiety, depression, and loneliness, and often adopt negative coping styles to deal with things (Kong and Zhang, 2007). The convenience and entertainment of smartphones can be used to vent bad experiences, prompting individuals to use smartphones more frequently to relieve their negative emotions, in happiness (Lepp et al., 2014). Meanwhile, as virtual communication reduces face-to-face communication, excessive use of smartphones will reduce the quality of in-person interaction (Rotondi et al., 2017), thus affecting the satisfaction that individuals derive from social relationships (Satici and Deniz, 2020), and reducing their subjective well-being.

### Moderating effects

Age significantly moderated the relationship between SA and positive emotions. The effect on undergraduates was significantly higher than that on middle school students. The main reason for these differences was that college students’ availability, holding rate, and use frequency of smartphones was higher than that of middle school students (Yen et al., 2009), and they have a more serious tendency to virtualize realistic interpersonal communication (Zhou et al., 2011). When psychological distress occurs, they find it easier to escape and compensate with the help of smartphones and are also more likely to rely on them for solace (Kardefelt-Winther, 2014b). In turn, the dependence on smartphones further squeezes their real social interaction time and adversely affects their interpersonal relationships in reality. Additionally, good interpersonal relationships are an important source of positive emotions (Diener et al., 2010), which may lead to fewer positive emotions among college students. In addition, the number of middle school students in this study was small in the included studies (*k* = 1). Therefore, the results of this study cannot fully reflect the relationship between SA and positive emotions in different age groups. The results of this study need to be confirmed by further studies.

The tools for measuring SA significantly moderated the relationship between SA and alexithymia, attachment anxiety, and subjective well-being. First, in terms of alexithymia and attachment anxiety, MPATS (Xiong et al., 2012) (*r* = 0.51; *r* = 0.52, respectively) had the highest effect. This may be due to the different perspectives of the MPATS and other scales. The MPATS is based on the subjective experience of smartphone users’ social interactions. Moreover, individuals with higher levels of alexithymia and attachment anxiety have poor interpersonal adaptability in reality and experience higher social anxiety, but they still have strong social desire (Wastell and Taylor, 2002; Zhu et al., 2019), which makes them tend to establish contact with the outside world through mobile networks and other media to obtain a sense of intimacy. This eventually leads to the tendency of SA, resulting in a higher correlation, when using the MPATS. Second, in terms of subjective well-being, the MPAI (Leung, 2008) (*r* = –0.42) had the highest effect. This may be because MPAI mainly focuses on describing the impact of smartphones on users’ behavior and impairment of social functions. Subjective well-being is an individual’s overall evaluation of life conditions; therefore, the MPAI shows a higher correlation.

The tools used to measure subjective well-being significantly moderated the relationship between SA and subjective well-being. GWB (Duan, 1996) (*r* = –0.41) had the highest effect. This may be because GWB has a large number of items (33 in total) that can reflect individual subjective well-being more comprehensively and accurately. Other scales, such as IWB (Campbell, 1976), have only nine items. Although they can reflect the subjective well-being of individuals to a certain extent, some necessary information is inevitably lost. In addition, it may also be because the GWB scale used in this study was revised by Chinese scholars on Fazio’s general well-being schedule (Fazio, 1977), in combination with the economic and cultural characteristics of their own countries. Most of the studies included in this meta-analysis were Chinese samples; therefore, the correlation coefficient measured by GWB was relatively high.

The tools used to measure negative emotions significantly moderated the relationship between SA and negative emotions. The PANAS (Watson et al., 1988) (*r* = 0.37) had the highest effect. This may be because of the different test contents and dimensions of each scale. PANAS includes two emotional dimensions: positive and negative. The two dimensions contained ten items each. The SWB scale (Yan et al., 2003) includes four dimensions: overall subjective well-being, life satisfaction, positive emotion, and negative emotion. Positive and negative emotions contain six and eight items, respectively. Therefore, PANAS is closer to the two-dimensional essence of emotion, and hence the correlation between them reflected by the PANAS scale is greater.

### Study implications

This study is of great significance for the prevention and intervention of SA. First, the results described the correlation between SA and alexithymia, insecure attachment styles, and subjective well-being, which can provide a reference for future studies. This means that to reduce the negative impact of SA on individuals, we need to not only improve the level of subjective well-being of individuals but also pay attention to timely screening and to identify individuals with alexithymia and insecure attachment styles. At the same time, researchers should further develop effective strategies (e.g., mindfulness), starting with individual emotional training, so that individuals can master emotional types, understand emotional characteristics, and alleviate the negative effects of alexithymia on SA by enhancing their ability to identify and describe emotions (Gao T. et al., 2018; Li and Hao, 2019). In addition, most studies have shown that mindfulness can significantly improve subjective well-being and life satisfaction, enhance positive emotions, and reduce negative emotions (Shapiro et al., 2007; Kieviet-Stijnen et al., 2008; Amundsen et al., 2020). In addition, researchers can use psychological counseling and treatment programs such as group therapy (Yuchang et al., 2017) to focus on attachment construction and help smartphone addicts establish healthy attachment relationships and secure attachment styles. Second, there was no significant difference between genders in SA problems accompanied by alexithymia, insecure attachment styles, and low subjective well-being. In future interventions, it is important to pay attention to the comprehensiveness of group coverage. Third, age significantly moderated the relationship between SA and positive emotion. This can remind parents and educators that it is necessary to pay attention to the psychological states of college students in time, and that individuals can have more positive emotions by organizing regular physical exercise. It is worth noting that owing to the small number of middle school students in the studies included in the meta-analysis, this conclusion needs to be further verified. Fourth, the SA measurement tools significantly moderated the relationship between SA and alexithymia, attachment anxiety, and subjective well-being. This may invite researchers and clinicians to use common criteria to define SA whenever possible, to reduce potential differences. Finally, there are differences in the predictive power of various subjective well-being measurement tools, which informs researchers to choose a scale with a more comprehensive measurement and higher fit when using subjective well-being measuring tools in the future, rather than just considering the brevity of the number of items on the scale. The internal validity of the measurement of a scale that is too concise is reduced. Based on this result, GWB and PANAS are good choices for future studies.

## Limitations and prospects

Previous studies on the relationship between SA and alexithymia, attachment style, and subjective well-being have been inconsistent. In this study, the meta-analysis was used to investigate the relationship between SA and alexithymia, attachment style, and subjective well-being, and to clarify the controversy about the size of the correlation between them in the empirical study. However, this study also has some limitations. First, the data of this study were collected through a questionnaire survey; therefore, information bias and reporting bias are inevitable, and more objective forms can be considered for future collection. Second, the studies included in this meta-analysis mainly focused on students. In the future, the subject group can be further expanded to explore whether there are differences in the relationship between SA and alexithymia, attachment style, and subjective well-being among diverse subject groups. Finally, although our goal was to identify studies carried out worldwide, most of the studies included were samples from Asian countries. This limited sample size restricts the universality of the current findings, and these relationships can be investigated in a broader national and cultural context in the future.

## Conclusion

The current meta-analysis found that SA was highly positively correlated with alexithymia, attachment anxiety, and negative emotions; lowly positively with attachment avoidance; highly negatively with subjective well-being; and lowly negatively correlated with life satisfaction and positive emotions. Therefore, in SA prevention and intervention, more attention should be paid to individuals with high levels of alexithymia, insecure attachment, and negative emotions. We need to not only pay attention to the cultivation of emotional ability and the construction of secure attachment patterns but also help them improve their subjective well-being in daily life and study, learn to use smartphones reasonably, and avoid the harm of addiction.

## Data availability statement

The original contributions presented in this study are included in the article/Supplementary material, further inquiries can be directed to the corresponding author/s.

## Author contributions

YD, HH, and CC conceived and designed the study. YD, HH, YZ, CC, HW, and JY contributed to the data curation, software, and formal analysis. YD and HH wrote the manuscript. YD, HH, YZ, QP, JY, GL, HW, and CC revised the manuscript. HW and CC contributed to the funding acquisition and supervision. All authors approved the final manuscript to be published.
