# Supplementary material for: Correlations between smartphone addiction and alexithymia, attachment style, and subjective well-being: A meta-analysis
Source: Front Psychol. 2022 Sep 2;13:971735. doi: 10.3389/fpsyg.2022.971735 (PMC9481561; doi:10.3389/fpsyg.2022.971735)
Supplement: Supplementary file 2 [file Table_2.DOCX]

**Appendix B search strategy**

***PubMed***

#1: “Cell Phone”[Mesh]

#2: ((((((((mobile phone[Title/Abstract]) OR (smart phone[Title/Abstract])) OR (smartphone[Title/Abstract])) OR (Cellular phone[Title/Abstract])) OR (transportable Cellular Phones[Title/Abstract])) OR (portable Cellular Phone[Title/Abstract])) OR (Cellular Telephone[Title/Abstract])) OR (Mobile Telephone[Title/Abstract])) OR (Car Phone[Title/Abstract])

#3: #1 or #2

#4: “Behavior, Addictive”[Mesh]

#5: (((((((addiction[Title/Abstract]) OR (dependence[Title/Abstract])) OR (abuse[Title/Abstract])) OR (dependency[Title/Abstract])) OR (addicted to[Title/Abstract])) OR (overuse[Title/Abstract])) OR (problem use[Title/Abstract])) OR (compensatory use[Title/Abstract])

#6: #4 or #5

#7: "Affective Symptoms"[Mesh]

#8: ((((((((Affective Symptom[Title/Abstract]) OR (Symptom, Affective[Title/Abstract])) OR (Symptoms, Affective[Title/Abstract])) OR (Alexithymia[Title/Abstract])) OR (Alexithymias[Title/Abstract])) OR (Emotional Disturbances[Title/Abstract])) OR (Disturbance, Emotional[Title/Abstract])) OR (Disturbances, Emotional[Title/Abstract])) OR (Emotional Disturbance[Title/Abstract])

#9: #7 or #8

#10: "Reactive Attachment Disorder"[Mesh]

#11: ((((Attachment[Title/Abstract]) OR (Attachment Disorder, Reactive[Title/Abstract])) OR (Attachment Disorders, Reactive[Title/Abstract])) OR (Disorder, Reactive Attachment[Title/Abstract])) OR (Disorders, Reactive Attachment[Title/Abstract])

#12: #10 or #11

#13: (((((happiness[Title/Abstract]) OR (well-being[Title/Abstract])) OR (subjective well-being[Title/Abstract])) OR (life satisfaction[Title/Abstract])) OR (positive emotion[Title/Abstract])) OR (negative emotion[Title/Abstract])

#14: #3 and #6 and #9

#15: #3 and #6 and #12

#16: #3 and #6 and #13

***Web of science***

((TS=(“Cell Phone” OR “mobile phone” OR “smart phone” OR smartphone OR “Cellular phone” OR “transportable Cellular Phones” OR “portable Cellular Phone” OR “Cellular Telephone” OR “Mobile Telephone” OR “Car Phone”)) AND TS=(“Behavior, Addictive” OR addiction OR dependence OR abuse OR dependency OR “addicted to” OR overuse OR “problem use” OR “compensatory use”)) AND TS=(“Affective Symptoms” OR “Affective Symptom” OR “Symptom, Affective” OR “Symptoms, Affective” OR Alexithymia OR Alexithymias OR “Emotional Disturbances” OR “Disturbance, Emotional” OR “Disturbances, Emotional” OR “Emotional Disturbance”)

Timespan: All years

TS=(“Cell Phone” OR “mobile phone” OR “smart phone” OR smartphone OR “Cellular phone” OR “transportable Cellular Phones” OR “portable Cellular Phone” OR “Cellular Telephone” OR “Mobile Telephone” OR “Car Phone”) AND TS=(“Behavior, Addictive” OR addiction OR dependence OR abuse OR dependency OR “addicted to” OR overuse OR “problem use” OR “compensatory use”) AND TS=(“Reactive Attachment Disorder” OR “Attachment” OR “Attachment Disorder, Reactive” OR “Attachment Disorders, Reactive” OR “Disorder, Reactive Attachment” OR “Disorders, Reactive Attachment”)

Timespan: All years

(((TS=(“Cell Phone” OR “mobile phone” OR “smart phone” OR smartphone OR “Cellular phone” OR “transportable Cellular Phones” OR “portable Cellular Phone” OR “Cellular Telephone” OR “Mobile Telephone” OR “Car Phone”)) AND TS=(“Behavior, Addictive” OR addiction OR dependence OR abuse OR dependency OR “addicted to” OR overuse OR “problem use” OR “compensatory use”))) AND TS=(happiness OR well-being OR "subjective well-being" OR "life satisfaction" OR "positive emotion" OR "negative emotion")

Timespan: All years

***Embase***

#1. 'mobile phone'/exp

#2. 'cell phone':ab,ti

#3. 'smart phone':ab,ti

#4. 'smartphone':ab,ti

#5. 'cellular phone':ab,ti

#6. 'transportable cellular phones':ab,ti

#7. 'portable cellular phone':ab,ti

#8. 'cellular telephone':ab,ti

#9. 'mobile telephone':ab,ti

#10. 'car phone':ab,ti

#11. #1 OR #2 OR #3 OR #4 OR #5 OR #6 OR #7 OR #8 OR #9 OR #10

#12. 'addiction'/exp

#13. 'behavior, addictive':ab,ti

#14. 'dependence':ab,ti

#15. 'abuse':ab,ti

#16. 'dependency':ab,ti

#17. 'addicted to':ab,ti

#18. 'overuse':ab,ti

#19. 'problem use':ab,ti

#20. 'compensatory use':ab,ti

#21. #12 OR #13 OR #14 OR #15 OR #16 OR #17 OR #18 OR #19 OR #20

#22. 'alexithymia'/exp

#23. 'affective symptoms':ab,ti

#24. 'affective symptom':ab,ti

#25. 'symptom, affective':ab,ti

#26. 'symptoms, affective':ab,ti

#27. 'alexithymias':ab,ti

#28. 'emotional disturbances':ab,ti

#29. 'emotional disturbance':ab,ti

#30. 'disturbance, emotional':ab,ti

#31. 'disturbances, emotional':ab,ti

#32. #22 OR #23 OR #24 OR #25 OR #26 OR #27 OR #28 OR #29 OR #30 OR #31

#33. #11 AND #21 AND #32

#34. 'reactive attachment disorder':ab,ti

#35. 'attachment disorder, reactive':ab,ti

#36. 'attachment disorders, reactive':ab,ti

#37. 'disorder, reactive attachment':ab,ti

#38. 'disorders, reactive attachment':ab,ti

#39. 'attachment':ab,ti

#40. 'reactive attachment disorders':ab,ti

#41. #34 OR #35 OR #36 OR #37 OR #38 OR #39 OR #40

#42. #11 AND #21 AND #41

#43. 'happiness':ab,ti

#44. 'well-being':ab,ti

#45. 'subjective well-being':ab,ti

#46. 'life satisfaction':ab,ti

#47. 'positive emotion':ab,ti

#48. 'negative emotion':ab,ti

#49. #43 OR #44 OR #45 OR #46 OR #47 OR #48

#50. #11 AND #21 AND #49

***PsycINFO and PsycArticles***

Boolean/Phrase: (“Cell Phone” OR “mobile phone” OR “smart phone” OR smartphone OR “Cellular phone” OR “transportable Cellular Phones” OR “portable Cellular Phone” OR “Cellular Telephone” OR “Mobile Telephone” OR “Car Phone”) AND (“Behavior, Addictive” OR addiction OR dependence OR abuse OR dependency OR “addicted to” OR overuse OR “problem use” OR “compensatory use”) AND (“Reactive Attachment Disorder” OR “Attachment” OR “Attachment Disorder, Reactive” OR “Attachment Disorders, Reactive” OR “Disorder, Reactive Attachment” OR “Disorders, Reactive Attachment”)

Limiters: Language – English

Timespan: All years

Boolean/Phrase: (“Cell Phone” OR “mobile phone” OR “smart phone” OR smartphone OR “Cellular phone” OR “transportable Cellular Phones” OR “portable Cellular Phone” OR “Cellular Telephone” OR “Mobile Telephone” OR “Car Phone”) AND (“Behavior, Addictive” OR addiction OR dependence OR abuse OR dependency OR “addicted to” OR overuse OR “problem use” OR “compensatory use”) AND (“Reactive Attachment Disorder” OR “Attachment” OR “Attachment Disorder, Reactive” OR “Attachment Disorders, Reactive” OR “Disorder, Reactive Attachment” OR “Disorders, Reactive Attachment”)

Limiters: Language – English

Timespan: All years

Boolean/Phrase: (“Cell Phone” OR “mobile phone” OR “smart phone” OR smartphone OR “Cellular phone” OR “transportable Cellular Phones” OR “portable Cellular Phone” OR “Cellular Telephone” OR “Mobile Telephone” OR “Car Phone”) AND (“Behavior, Addictive” OR addiction OR dependence OR abuse OR dependency OR “addicted to” OR overuse OR “problem use” OR “compensatory use”) AND (happiness OR well-being OR "subjective well-being" OR "life satisfaction" OR "positive emotion" OR "negative emotion")

Limiters: Language – English

Timespan: All years
