# Supplementary material for: Correlations between smartphone addiction and alexithymia, attachment style, and subjective well-being: A meta-analysis
Source: Front Psychol. 2022 Sep 2;13:971735. doi: 10.3389/fpsyg.2022.971735 (PMC9481561; doi:10.3389/fpsyg.2022.971735)
Supplement: Supplementary file 3 [file Table_3.DOCX]

**Appendix C.** Quality assessment for the 110 studies in the current meta-analysis.

| Study | Quality Item | | | | | | | | | |
| --- | --- | --- | --- | --- | --- | --- | --- | --- | --- | --- |
|  | Item1 | Item2 | Item3 | Item4 | Item5 | Item6 | Item7 | Item8 | Item9 | Total |
| Ge X(2013)(84) | Y | Y | Y | Y | Y | N | Y | Y | Y | 8 |
| Wang H(2014)(85) | Y | Y | Y | Y | Y | N | Y | Y | Y | 8 |
| Huang H(2014)(86) | Y | Y | Y | Y | Y | Y | Y | Y | Y | 9 |
| Ji J(2014)(87) | Y | N | Y | N | Y | N | Y | Y | Y | 6 |
| Yuan W(2014)(88) | Y | Y | Y | Y | Y | Y | U | Y | Y | 8 |
| Zhang J(2015)(89) | Y | N | Y | N | Y | Y | Y | Y | Y | 7 |
| Zeng Y(2015)(90) | Y | N | Y | N | Y | Y | Y | Y | Y | 7 |
| Zhang J(2015)(91) | Y | Y | Y | Y | Y | N | Y | Y | Y | 8 |
| Deng Z(2015)(92) | Y | N | Y | Y | Y | Y | Y | Y | Y | 8 |
| Kan J(2015)(93) | Y | Y | Y | Y | Y | Y | U | Y | Y | 8 |
| Tang Y(2015)(94) | Y | Y | Y | Y | Y | N | U | Y | Y | 7 |
| Wang Y(2015)(95) | Y | Y | Y | Y | Y | Y | Y | Y | Y | 9 |
| Xie Y(2015)(96) | Y | Y | Y | Y | Y | N | Y | Y | Y | 8 |
| Hou R(2016)(97) | Y | Y | Y | Y | Y | N | Y | Y | Y | 8 |
| Zheng J(2016)(98) | Y | Y | Y | N | Y | Y | Y | Y | Y | 8 |
| Li J(2016)(99) | Y | N | Y | Y | Y | Y | Y | Y | Y | 8 |
| Xie F(2016)(100) | Y | U | Y | Y | Y | Y | Y | Y | Y | 8 |
| Du J(2016)(41) | Y | Y | Y | Y | Y | N | Y | Y | Y | 8 |
| Ge X(2016)(101) | Y | Y | Y | Y | Y | N | U | Y | Y | 7 |
| Li C(2016)(102) | Y | Y | Y | Y | Y | N | Y | Y | Y | 8 |
| Samaha M(2016)(103) | Y | Y | Y | Y | Y | Y | Y | Y | Y | 9 |
| Sun J(2017)(104) | Y | N | Y | N | Y | Y | U | Y | Y | 6 |
| Han L(2017)(38) | Y | Y | Y | N | Y | N | Y | Y | Y | 7 |
| Arpaci I(2017)(105) | Y | N | Y | Y | Y | N | Y | Y | N | 6 |
| Jin Y(2017)(106) | Y | U | Y | Y | Y | Y | Y | Y | Y | 8 |
| Li X(2017)(107) | Y | Y | Y | Y | Y | Y | Y | Y | Y | 9 |
| Li Z(2017)(49) | Y | Y | Y | Y | Y | N | N | Y | Y | 7 |
| Liu Q(2017)(108) | Y | N | Y | Y | Y | N | Y | Y | Y | 7 |
| Ouyang L(2017)(109) | Y | Y | Y | Y | Y | Y | Y | Y | Y | 9 |
| Peng Q(2017)(110) | Y | N | Y | Y | Y | N | U | Y | Y | 6 |
| Wang F(2017)(111) | Y | Y | Y | N | Y | N | U | Y | Y | 6 |
| Zhang B(2017)(112) | Y | Y | Y | Y | Y | Y | Y | Y | Y | 9 |
| Hao C(2018)(113) | Y | Y | Y | Y | Y | Y | U | Y | Y | 8 |
| Zhang Y(2018)(68) | Y | N | Y | Y | Y | N | Y | Y | Y | 7 |
| Gao T(2018)(28) | Y | N | Y | Y | Y | N | Y | Y | Y | 7 |
| Mei S(2018)(114) | Y | N | Y | Y | Y | N | Y | Y | Y | 7 |
| Huang H(2018)(115) | Y | Y | Y | Y | Y | Y | N | Y | Y | 8 |
| Wang Y(2018)(42) | Y | U | Y | Y | Y | Y | N | Y | Y | 7 |
| Kim E(2018)(40) | Y | N | Y | Y | Y | N | Y | Y | Y | 7 |
| Gao Y(2018)(62) | Y | Y | Y | Y | Y | Y | Y | Y | Y | 9 |
| Niu L(2018)(116) | Y | N | Y | Y | Y | N | Y | Y | Y | 7 |
| Ren X(2018)(117) | Y | N | Y | N | Y | N | Y | Y | Y | 6 |
| Xiong S(2018)(118) | Y | Y | Y | Y | Y | N | Y | Y | Y | 8 |
| Yang X(2018)(119) | Y | N | Y | Y | Y | N | U | Y | Y | 6 |
| Zhang Y(2018)(120) | Y | U | Y | Y | Y | N | Y | Y | Y | 7 |
| Zufeiya T(2018)(121) | Y | Y | Y | Y | Y | N | Y | Y | Y | 8 |
| Ozdemir B (2018)(48) | Y | N | Y | Y | Y | N | U | Y | Y | 6 |
| Aruna (2019)(122) | Y | N | Y | N | Y | Y | Y | Y | Y | 7 |
| Chen Y(2019)(123) | Y | Y | Y | Y | Y | Y | Y | Y | Y | 9 |
| Huang M (2019)(124) | Y | Y | Y | Y | Y | N | Y | Y | Y | 8 |
| Lin R(2019)(125) | Y | U | Y | Y | Y | Y | Y | Y | Y | 8 |
| Li X(2019)(126) | Y | U | Y | N | Y | N | Y | Y | Y | 6 |
| Hao Z(2019)(127) | Y | Y | Y | Y | Y | Y | Y | Y | Y | 9 |
| Yavuz M(2019)(29) | Y | Y | Y | Y | Y | Y | Y | Y | Y | 9 |
| Xu M(2019)(128) | Y | Y | Y | N | Y | Y | U | Y | Y | 7 |
| Yan D(2019)(129) | Y | Y | Y | N | Y | N | Y | Y | Y | 7 |
| Zhu H(2019)(130) | Y | N | Y | Y | Y | Y | Y | Y | Y | 8 |
| Liu Q(2019)(37) | Y | U | Y | N | Y | N | Y | Y | Y | 6 |
| An X(2019)(55) | Y | N | Y | Y | Y | Y | Y | Y | Y | 8 |
| Chen Y(2019)(51) | Y | N | Y | Y | Y | N | Y | Y | Y | 7 |
| Li S(2019)(54) | Y | U | Y | N | Y | Y | N | Y | Y | 6 |
| Tong Y(2019)(63) | Y | N | Y | Y | Y | N | Y | Y | Y | 7 |
| Yang L(2019)(57) | Y | U | Y | Y | Y | Y | Y | Y | Y | 8 |
| Yang (2019)(131) | Y | U | Y | N | Y | Y | Y | Y | Y | 7 |
| Zhang L(2019)(61) | Y | Y | Y | N | Y | Y | N | Y | Y | 7 |
| Zhao X(2019)(132) | Y | N | Y | Y | Y | N | U | Y | Y | 6 |
| Horwood (2019)(59) | Y | Y | Y | Y | Y | N | Y | Y | Y | 8 |
| Md Nordin (2019)(60) | Y | N | Y | Y | Y | Y | Y | Y | Y | 8 |
| Song S(2019)(133) | Y | N | Y | Y | Y | N | Y | Y | N | 6 |
| Volkmer SA(2019)(46) | Y | U | Y | Y | Y | Y | Y | Y | Y | 8 |
| Yang Z(2019)(58) | Y | N | Y | Y | Y | N | Y | Y | Y | 7 |
| Eksi F(2020)(134) | Y | N | Y | Y | Y | Y | U | Y | Y | 7 |
| Huang M(2020)(135) | Y | Y | Y | Y | Y | N | Y | Y | Y | 8 |
| Yu P(2020)(136) | Y | U | Y | Y | Y | N | Y | Y | Y | 7 |
| Yu H (2020)(137) | Y | Y | Y | Y | Y | N | Y | Y | Y | 8 |
| Yuan M(2020)(138) | Y | Y | Y | N | Y | Y | Y | Y | Y | 8 |
| Elkholy H(2020)(139) | Y | U | Y | Y | Y | Y | Y | Y | Y | 8 |
| Hao Z(2020)(140) | Y | Y | Y | Y | Y | N | Y | Y | Y | 8 |
| Hao Z(2020)(141) | Y | Y | Y | Y | Y | N | Y | Y | Y | 8 |
| Remondi C(2020)(19) | Y | N | Y | Y | Y | N | Y | Y | N | 6 |
| Park I(2020)(36) | Y | N | Y | Y | Y | Y | Y | Y | Y | 8 |
| Li WF(2020)(142) | Y | U | Y | Y | Y | N | Y | Y | Y | 7 |
| Chen X(2020)(143) | Y | N | Y | Y | Y | N | Y | Y | N | 6 |
| Hu J(2020)(144) | Y | Y | Y | N | Y | Y | U | Y | Y | 7 |
| Liang J(2020)(145) | Y | N | Y | Y | Y | Y | Y | Y | Y | 8 |
| Liu Y(2020)(146) | Y | Y | Y | N | Y | N | Y | Y | Y | 7 |
| Xiao M(2020)(147) | Y | N | Y | Y | Y | Y | Y | Y | N | 7 |
| Zhang F(2020)(50) | Y | Y | Y | N | Y | Y | Y | Y | Y | 8 |
| Zhang Y(2020)(148) | Y | Y | Y | Y | Y | N | Y | Y | Y | 8 |
| Jeong YJ(2020)(149) | Y | U | Y | Y | Y | Y | Y | Y | N | 7 |
| Peng S(2020)(150) | Y | U | Y | Y | Y | Y | Y | Y | Y | 8 |
| Gao Q(2020)(53) | Y | Y | Y | Y | Y | Y | Y | Y | Y | 9 |
| Kaya A(2020)(151) | Y | U | Y | Y | Y | N | U | Y | Y | 6 |
| Satici B(2020)(20) | Y | U | Y | Y | Y | N | Y | Y | Y | 7 |
| Hou Y(2021)(56) | Y | Y | Y | Y | Y | N | Y | Y | Y | 8 |
| Sun C(2021)(152) | Y | Y | Y | Y | Y | Y | Y | Y | Y | 9 |
| Zhang C(2021)(153) | Y | Y | Y | Y | Y | N | U | Y | Y | 7 |
| Gündoğmuş I(2021)(27) | Y | Y | Y | Y | Y | Y | Y | Y | Y | 9 |
| Kaya B(2021)(30) | Y | Y | Y | Y | Y | N | Y | Y | Y | 8 |
| Xiao W(2021)(17) | Y | N | Y | Y | Y | Y | Y | Y | Y | 8 |
| Zhang CH(2021)(154) | Y | N | Y | Y | Y | N | Y | Y | Y | 7 |
| Gui Z(2021)(39) | Y | Y | Y | Y | Y | N | Y | Y | Y | 8 |
| Yao X(2021)(155) | Y | U | Y | N | Y | Y | Y | Y | Y | 7 |
| Bermingham L(2021)(18) | Y | N | Y | Y | Y | Y | Y | Y | Y | 8 |
| Parent N(2021)(35) | Y | N | Y | Y | Y | N | Y | Y | Y | 7 |
| Huang M(2021)(64) | Y | Y | Y | Y | Y | Y | U | Y | Y | 8 |
| Ding ZC(2021)(47) | Y | U | Y | Y | Y | N | Y | Y | Y | 7 |
| Li Y(2021)(156) | Y | Y | Y | Y | Y | N | Y | Y | Y | 8 |
| Wang C(2021)(52) | Y | N | Y | N | Y | Y | Y | Y | Y | 7 |
| Wang W(2021)(157) | Y | U | Y | Y | Y | N | Y | Y | Y | 7 |

*Abbreviations:* Y, yes; N, No; U, unclear.

Note: 1. Was the sample frame appropriate to address the target population?; 2. Were study participants sampled in an appropriate way?; 3. Was the sample size adequate?; 4. Were the study subjects and the setting described in detail?; 5. Was the data analysis conducted with sufficient coverage of the identified sample?; 6. Were valid methods used for the identification of the condition?; 7. Was the condition measured in a standard, reliable way for all participants?; 8. Was there appropriate statistical analysis?; 9. Was the response rate adequate, and if not, was the low response rate managed appropriately?
